# Supplementary material for: Conditional chemoconnectomics (cCCTomics) as a strategy for efficient and conditional targeting of chemical transmission
Source: eLife. 2024 Apr 30;12:RP91927. doi: 10.7554/eLife.91927 (PMC11060718; doi:10.7554/eLife.91927)
Supplement: Figure 3—source data 3. [file elife-91927-fig3-data3.zip › Figure 3 source data3 part 1.zip!/Figure 3-Source data 3-part1/1081-1-CRISPResso_Report_8zosk6yj.zip!/CRISPResso_on_1081_3/3a.Indel_size_distribution.pdf]

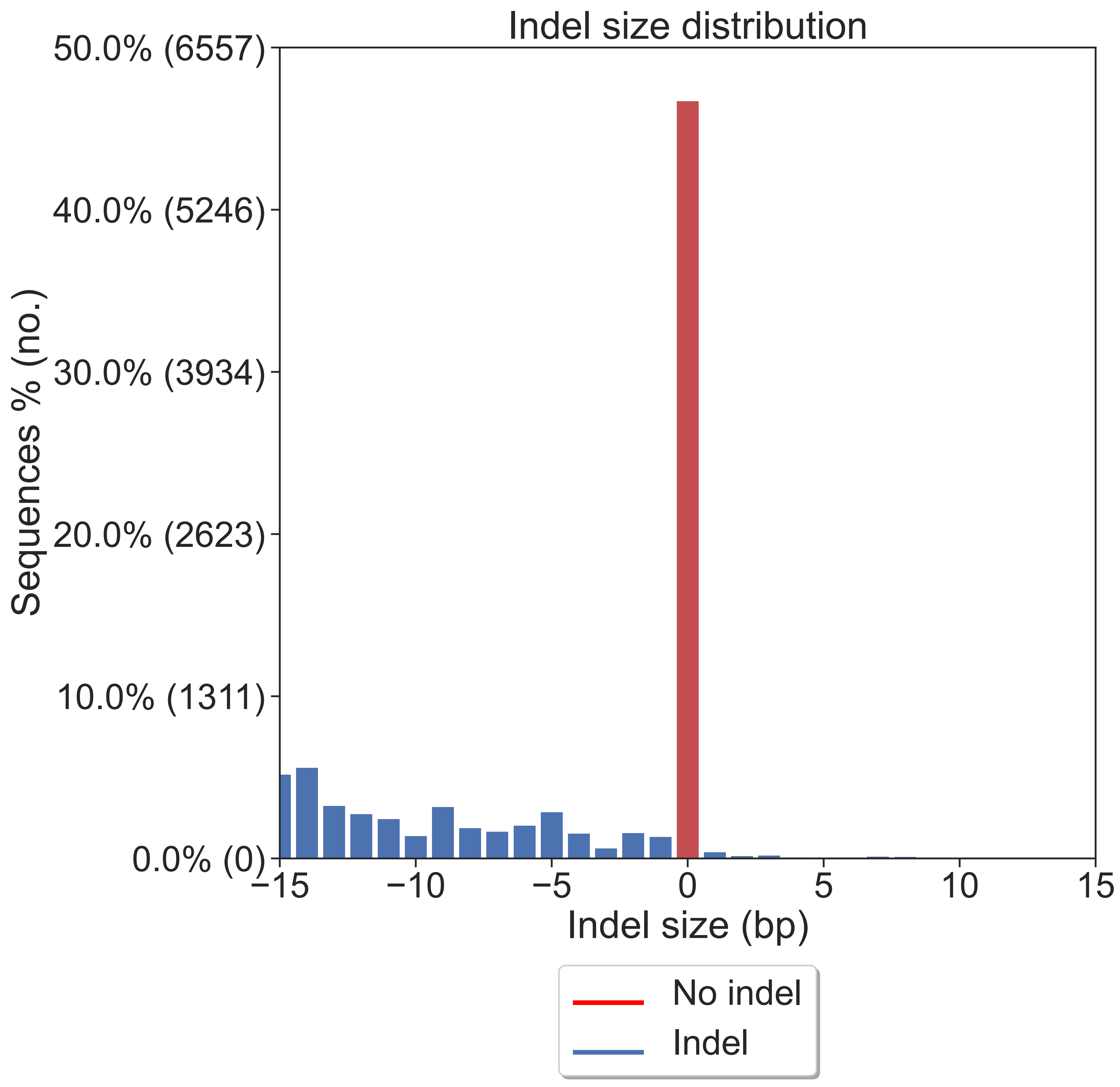

Indel size distribution
Sequences % (no.)
50.0% (6557)
40.0% (5246)
30.0% (3934)
20.0% (2623)
10.0% (1311)
0.0% (0)
−15
−10
−5
0
5
10
15
Indel size (bp)
No indel
Indel
